# High energy electron beam stimulated nanowelding of silver nanowire networks encapsulated with graphene for flexible and transparent electrodes

**DOI:** 10.1038/s41598-019-45887-5

**Published:** 2019-06-28

**Authors:** Su Jin Lee, Young Bum Lee, Yi Rang Lim, Jin Kyu Han, In Su Jeon, Garam Bae, Yeoheung Yoon, Wooseok Song, Sung Myung, Jongsun Lim, Ki-Seok An, Sun Sook Lee

**Affiliations:** 1 Thin Film Materials Research Center, Korea Research Institute of Chemical Technology, Yuseong Post Office Box 107, Daejeon, 305-600 Republic of Korea; 2 School of Electrical and Electronic Engineering, Yonsei University, 50 Yonsei-ro, Seodaemun-gu, Seoul, 03722 Republic of Korea; 3 Department of Energy Conversion and Storage, Technical University of Denmark, Frederiksborgvej 399, 4000 Roskilde, Denmark; 4 Department of Materials Science and Engineering, Hongik University, Seoul, 121-791 Republic of Korea; 5 Department of Physics, Sungkyunkwan University, Suwon, Gyeonggi-do 440-746 Republic of Korea

**Keywords:** Electronic properties and devices, Nanowires

## Abstract

Low-dimensional nanostructures and their complementary hybridization techniques are in the vanguard of technological advances for applications in transparent and flexible nanoelectronics due to the intriguing electrical properties related to their atomic structure. In this study, we demonstrated that welding of Ag nanowires (NWs) encapsulated in graphene was stimulated by flux-optimized, high-energy electron beam irradiation (HEBI) under ambient conditions. This methodology can inhibit the oxidation of Ag NWs which is induced by the inevitably generated reactive ozone as well as improve of their electrical conductivity. We have systematically explored the effects of HEBI on Ag NWs and graphene. The optimized flux for HEBI welding of the Ag NWs with graphene was 150 kGy, which decreased the sheet resistance of the graphene/Ag NWs to 12 Ohm/sq. Following encapsulation with graphene, the initial chemical states of the Ag NWs were well-preserved after flux-tuned HEBI, whereas graphene underwent local HEBI-induced defect generation near the junction area. We further employed resonant Raman spectroscopy to follow the structural evolution of the sacrificial graphene in the hybrid film after HEBI. Notably, the sheet resistance of the welded Ag NWs encapsulated with graphene after HEBI was well-maintained even after 85 days.

## Introduction

It is widely recognized that the prerequisites for the development of high-performance transparent electrodes are high electrical conductivity and high optical transparency. To date, indium tin oxide (ITO) has been typically adopted for multi-purpose transparent electrodes in solar cells, touch panels, and organic light emitting diode panels. However, ITO films possess limitations to their application in next-generation, flexible nanoelectronics because of their physical inflexibility and high cost. Due to their intriguing optical and electrical properties, promising candidates such as metallic nanowires (NWs) and low-dimensional carbon nanostructures including carbon nanotubes (CNTs) and graphene have been developed as replacements for ITO films to fabricate key components of next-generation nanoelectronics^1–9^. Unfortunately, the use of these materials in practical applications has been hampered. For example, two-dimensional monolayer graphene possesses excellent optical transmittance (~97.7%)^10^, however, its doping and layer-by-layer stacking, which are required to decrease its intrinsic sheet resistance (300–1000 Ohm/sq)^11^, are plagued by issues such as doping stability, the presence of residual carbon, and complicated procedures. One-dimensional conductors, including Ag NWs and CNTs, also encounter insurmountable hurdles involving high wire-to-wire junction resistance and low corrosion resistance^12^. To overcome these issues, the complementary hybridization of nanostructures, in terms of their properties and effective welding processes to decrease the junction resistance, are highly required^13^. In previous studies, welding processes for randomly networked metallic NWs with low sheet resistance have included plasma treatment, UV-pulsed laser irradiation, thermal annealing, and pressure welding^14–18^. However, these approaches suffer from limitations such as requirements of large-scale fabrication and low-temperature processes for the application of plastic substrates for flexible nanoelectronics. In this study, we firstly established a unique and facile methodology for welding Ag NWs using high-energy electron beam irradiation (HEBI) under ambient conditions over a large area (90 × 90 cm^2^). In general, the structural manipulation of carbon nanostructures via knock-on collisions of highly energetic electrons has been carried out using transmission electron microscopy (TEM) under high vacuum^19–21^. In previous studies, the decoration of CNTs with Au, Ni, and Pt nanoparticles and the hybridization of graphene with ZnO thin films were performed using MeV electron beam irradiation under ambient conditions, to tune their electrical properties^22–24^. Here, we rationally designed that the welding of Ag NWs encapsulated in graphene was stimulated by flux-optimized HEBI under ambient conditions to inhibit the oxidation of the Ag NWs by the inevitably generated reactive ozone, as well as to improve their electrical conductivity. This strategy enables the hybridization of graphene with the Ag NWs to provide an additional conducting path, and to simultaneously passivate the HEBI-induced oxidation of the Ag NWs, which was previously explored in a similar system (graphene on Cu NWs)^25^. As a result, long-term, air-stable Ag NWs-graphene hybrid conducting films with a low sheet resistance could be obtained. Additionally, an in-depth study of the structural and chemical features of graphene and the welded Ag NWs after flux-optimized HEBI was implemented.

## Results and Discussion

### Structural impacts of HEBI on the Ag NWs and the graphene/Ag NWs

**Figure 1:**
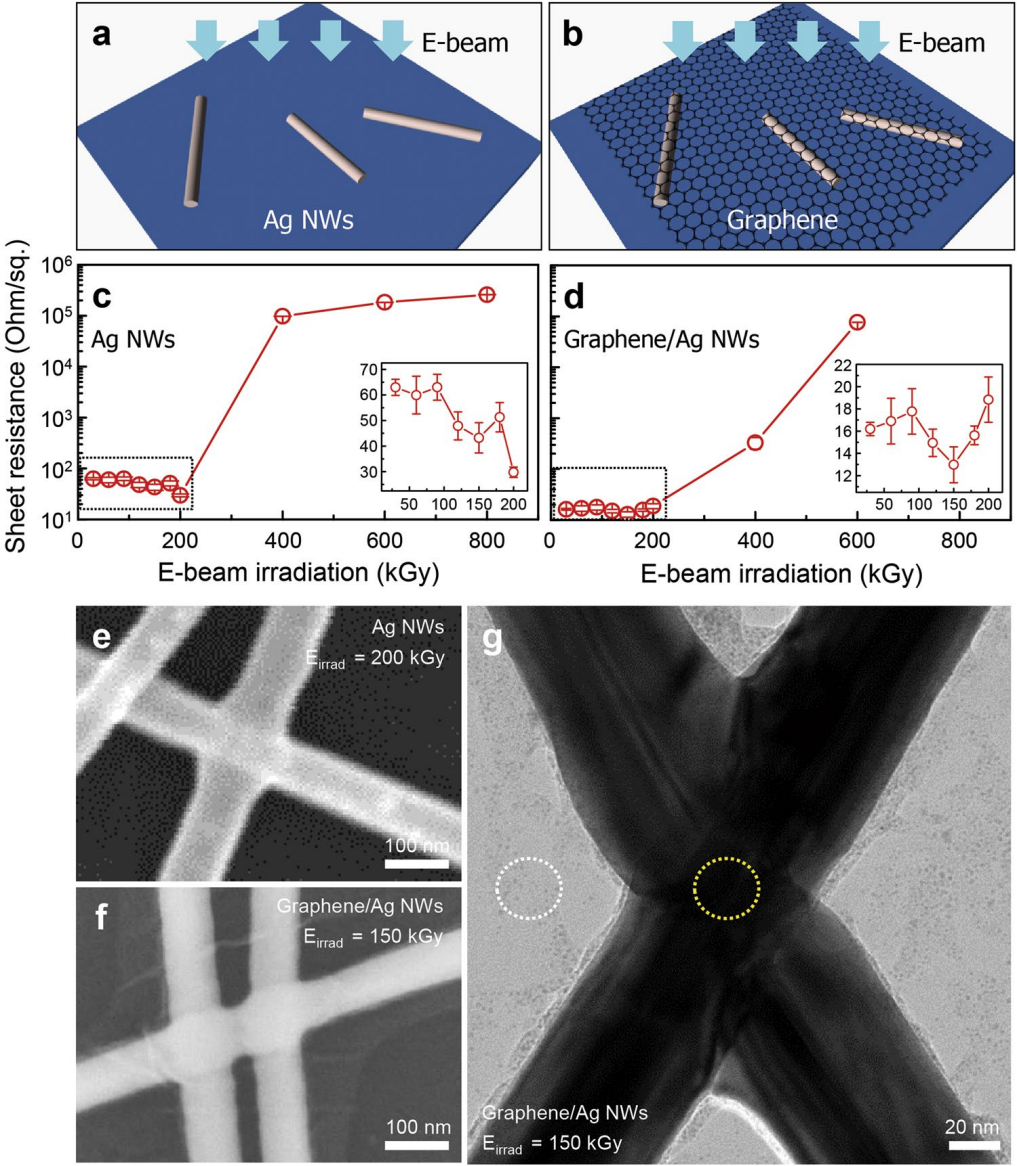
Structural chracterization of Ag NWs and graphene/welded Ag NWs after HEBI. (**a,b**) Schematic representation of the Ag NWs and the graphene/Ag NWs on SiO_2_/Si substrates treated with the HEBI. Plots of the sheet resistance for (**c**) the Ag NWs and (**d**) the graphene/Ag NWs as a function of the HEBI flux. Representative high-magnified SEM images of (**e**) the welded Ag NWs and (**f**) the graphene/welded Ag NWs after 1 MeV HEBI with a total flux of 150 kGy. (**g**) A TEM image of the graphene/welded Ag NWs after 1 MeV HEBI with a total flux of 150 kGy.

First, the effect of HEBI on the Ag NWs and the graphene/Ag NWs was explored, as depicted in Fig. 1a,b. Figure 1c,d represents plots of the sheet resistance for the Ag NWs and the graphene/Ag NWs as a function of the HEBI flux. Before HEBI, the initial sheet resistance values for the percolated networks of the Ag NWs and the graphene/Ag NWs correspond to 63 Ohm/sq and 16 Ohm/sq, respectively. This can be understood by the fact that the hybridization of graphene and Ag NWs can result in the formation of additional charge conducting paths, thus improving their electrical conductivity. Compared with the electrical properties of graphene-based hybrid nanomaterials, significantly higher electrical conductivity can be achieved in graphene/Ag NWs^26^. Interestingly, the lowest sheet resistance of Ag NWs/SiO_2_ is 29 Ohm/sq with a flux of 200 kGy, and that of graphene/Ag NWs/SiO_2_ is 12 Ohm/sq with a flux of 150 kGy, as shown in Fig. 1c,d. For HEBI with a flux of over 200 kGy, the sheet resistance values of both Ag NWs/SiO_2_ and graphene/Ag NWs/SiO_2_ increase significantly. Conversely, a negligible loss of the optical transmittance at 550 nm for the Ag NWs (83.0 ± 1.1%) and the graphene/Ag NWs (79.5 ± 1.1%) is observed after the flux-tunable HEBI (Supplementary Fig. S1). The sheet resistance and optical transmittance of previously-reported graphene/Ag NWs-based transparent electrodes are summarized (Supplementary Table S1), which signals that ther presented hybrid film has a competitive advantage over previously reported materials. The structural characterization of the Ag NWs/SiO_2_ and the graphene/Ag NWs/SiO_2_ was implemented to understand the reasons for the decreased sheet resistances under optimized irradiation conditions by scanning electron microscopy (SEM) and TEM. Figure 1e displays a representative SEM image of the Ag NWs/SiO_2_ with HEBI at a flux of 200 kGy, revealing that the two cross-stacked Ag NWs are welded unambiguously. For the graphene/Ag NWs/SiO_2_ with HEBI at a flux of 150 kGy, the Ag NWs are fully encapsulated in graphene and the formation of the wrinkle structure as well as electron-beam-stimulated welding of the Ag NWs can be observed in Fig. 1f. Additionally, the structure of the Ag NWs is well-preserved after HEBI (Supplementary Fig. S2). Based on these results, it can be concluded that HEBI-induced welding of the Ag NWs allows a decrease in the high wire-to-wire junction resistance, thus resulting in an extremely low sheet resistance. For HEBI with a flux of over 200 kGy, structural deformation occurs in the Ag NWs, however, the graphene in the hybrid sample inhibits structural damage in the Ag NWs (Supplementary Fig. S3). These results are strongly correlated with the sheet resistance of Ag NWs and graphene/Ag NWs with HEBI at a flux of 400 kGy. Figure 1g exhibits a representative TEM image of the welded Ag NWs with graphene induced by HEBI with a flux of 150 kGy, revealing that HEBI-induced welding of Ag NWs with graphene is elucidated. Selected area electron diffraction (SAED) patterns for the hybrid film from the graphene-only region and the welded Ag NWs with graphene region are displayed (Supplementary Fig. S4). The clear hexagonal diffraction pattern from the graphene-only region can be attributed to the presence of monolayer graphene. The diffraction pattern corresponding to the welded Ag NWs with graphene region is superimposed with hexagonal and rhombus structures, which are associated with the structure of the Ag NWs encapsulated in graphene. The intensity of the diffraction spots related with Ag NWs is greater than that of graphene. The reduction in the sheet resistance, induced by HEBI-stimulated Ag NWs welding, occurred at different irradiation fluxes for Ag NWs (200 kGy) and graphene/Ag NWs (150 kGy), presumably due to the fact that the heat generated at the junction induced by knock-on collisions of accelerated electrons was easily transferred by the excellent thermal conductivity of the graphene. Consequently, the reduction in the welding threshold can be attributed to the presence of graphene.

### Chemical indentification and electrical characterization of Ag NWs and graphene/welded Ag NWs

**Figure 2:**
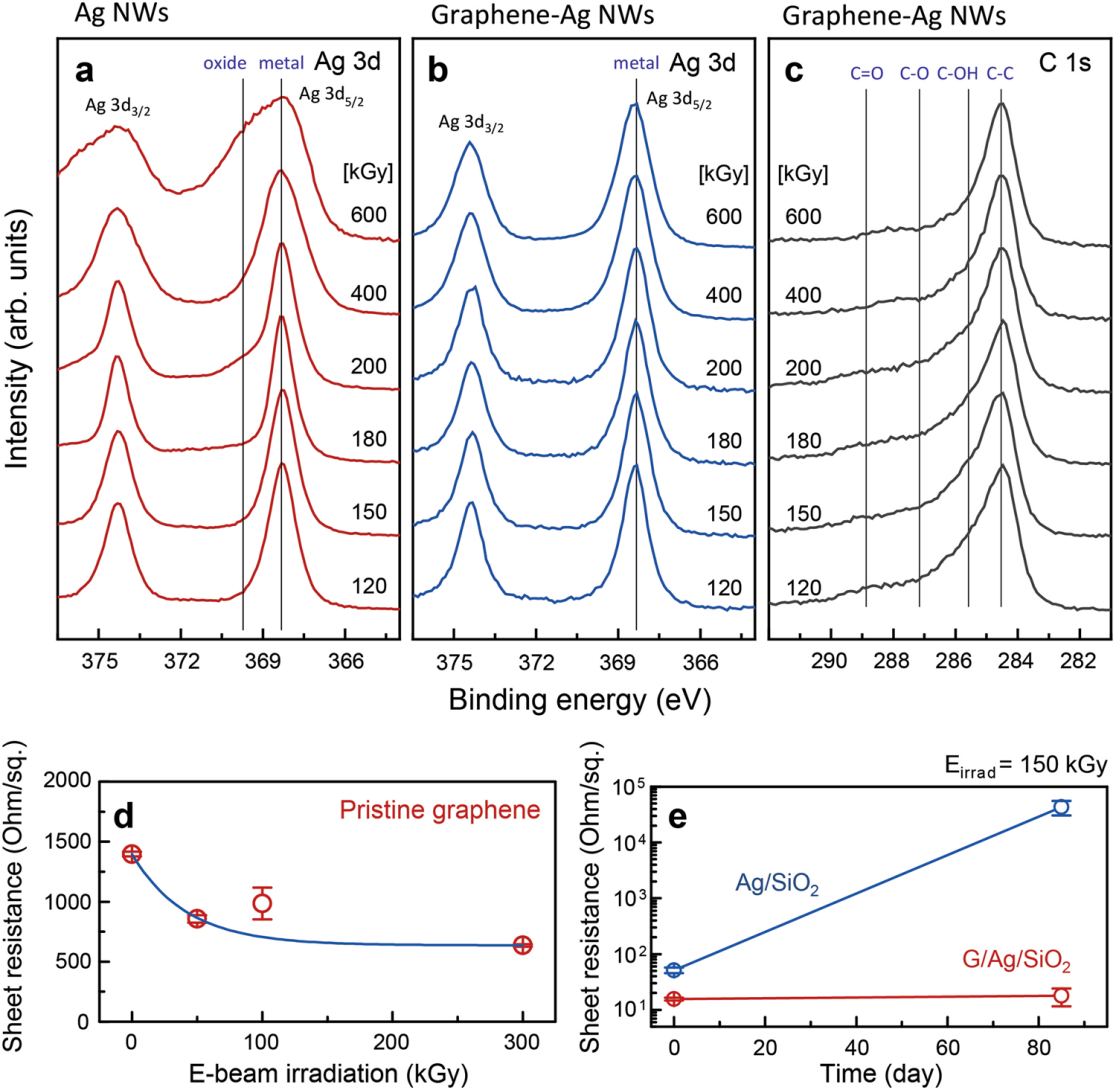
Chemical indentification and sheet resistance of Ag NWs and graphene/welded Ag NWs after HEBI. XPS Ag *3d* and C *1s* core level spectra for (**a**) the Ag NWs on SiO_2_ (300 nm)/Si(001), (**b,c**) the graphene/Ag NWs after 1 MeV HEBI with a total flux of 120–600 kGy. (**d**) Sheet resistance of pristine graphene as a function of HEBI total flux. (**e**) Long-term stability of the Ag/SiO_2_ and the graphene/Ag NWs/SiO_2_ after 1 MeV HEBI with a total flux of 150 kGy for 80 days.

The effects of flux-tunable HEBI on the chemical states of the Ag NWs and the graphene/Ag NWs hybrid films were explored by XPS. Figure 2a exhibits the Ag *3d* core level spectra of the Ag NWs/SiO_2_ after 1 MeV HEBI with a total flux of 120–600 kGy, revealing that the intensity of higher-binding energy oxidation states of the Ag *3d*_*5/2*_ and *3d*_*3/2*_ peaks increases with increasing HEBI flux. This result is strongly correlated with the variation in the sheet resistance of the Ag NWs after HEBI with a higher flux (>400 kGy). We mentioned earlier that HEBI was implemented under ambient conditions, whereby the oxidation of the Ag NWs occurs by the generation of reactive ozone. On the contrary, it should be noted that the initial chemical states of the Ag NWs encapsulated in graphene are well-preserved obviously, as shown in Fig. 2b. Based on these results, we suggest that the graphene acts a critical role as a protective layer, preventing the HEBI-induced oxidation of the Ag NWs. Due to encapsulation in graphene, the oxidation of the Ag NWs is effectively suppressed due to the excellent gas barrier property of graphene, which is in coincided with the results for sheet resistance. Figure 2c indicates the C *1s* core level spectra of the graphene/Ag NWs on SiO_2_ after HEBI with a total flux of 120–600 kGy, in which predominant *sp*^2^ C-C (E_B_ = 284.5 eV) and oxygen functional groups including C–OH, C–O, and C=O combined with PMMA-induced bonding states are observed. The formation of PMMA residue on the graphene surface is thought to be induced by the PMMA-mediated wet transfer process. Interestingly, with an increase in the total flux of the HEBI, the intensity of the PMMA-related peaks decreases gradually. This phenomenon can cause a reduction of the sheet resistance when increasing the total flux of the electron beam irradiating the pristine graphene, as displayed in Fig. 2d. In previous studies, inductively coupled plasma treatment, Ar ion beam irradiation, and laser beam cleaning were adopted to remove the PMMA residues from the graphene surfaces^27–29^. The long-term stability of sheet resistance for the Ag NWs and the graphene/Ag NWs on SiO_2_ after HEBI with a total flux of 150 kGy was confirmed by elucidating the protection effect by the gas barrier property of graphene, as seen in Fig. 2e. Notably, the sheet resistance of the HEBI-welded Ag NWs increases significantly from 51 Ohm/sq to 43,213 Ohm/sq, whereas that of the welded Ag NWs encapsulated in graphene is well-maintained from 15 Ohm/sq to 17 Ohm/sq after 85 days. Encapsulation of the welded Ag NWs in graphene allows for excellent long-term stability of the sheet resistance, which may be a solution to solve the problem of time-dependent deterioration of electrical conductivity in Ag NWs-based transparent electrodes.

### Structural evolution of graphene/welded Ag NWs after HEBI

**Figure 3:**
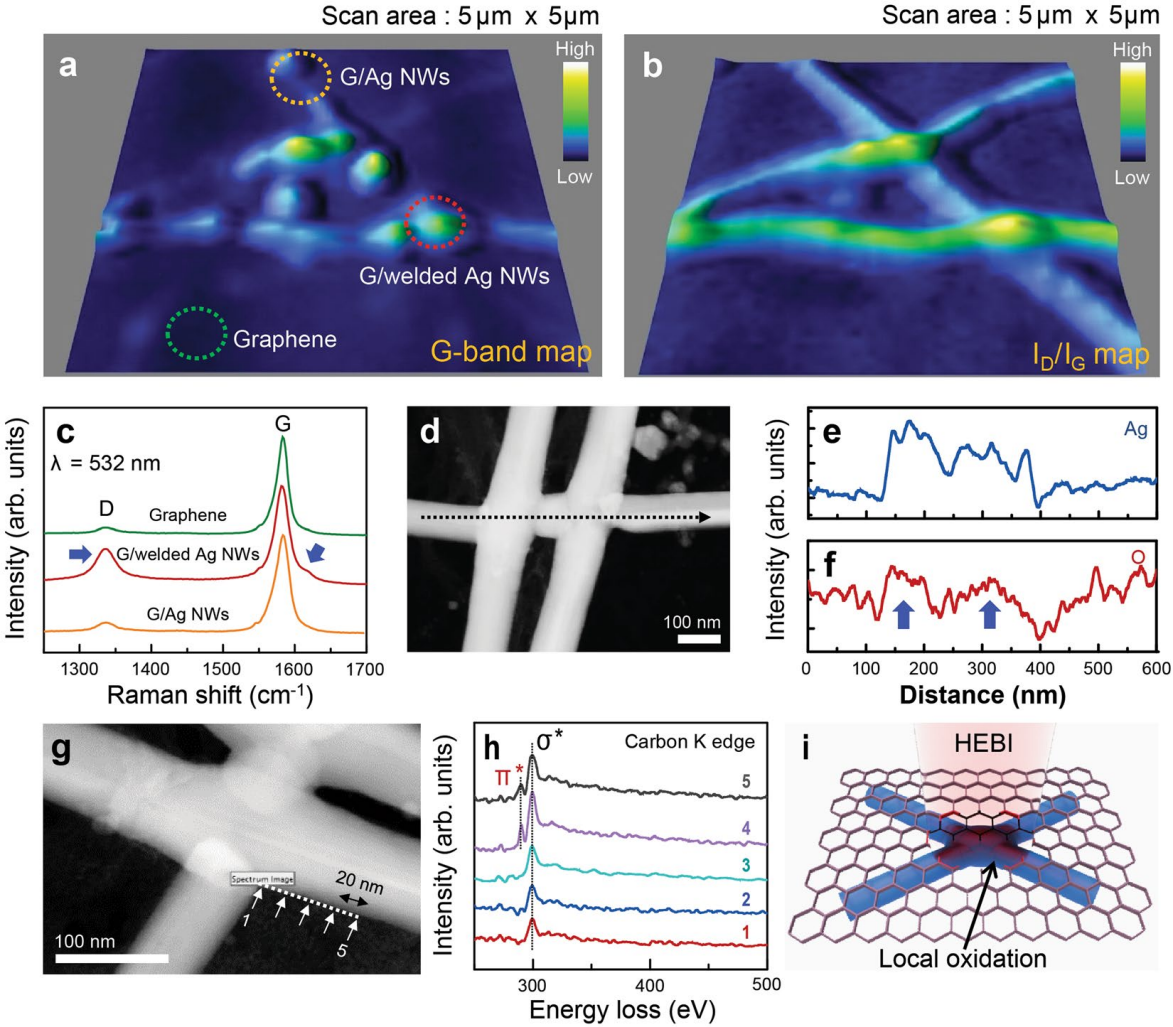
Structural evolution of the graphene/welded Ag NWs after HEBI. Raman maps recorded with an excitation wavelength of 532 nm of the (**a**) G-band and (**b**) I_D_/I_G_ for the graphene/Ag NWs after HEBI with a total flux of 150 kGy. (**c**) Raman spectra for the graphene-only region (green circle in **a**), the graphene/welded Ag NWs region (red circle), and the graphene/Ag NWs (yellow circle) region. (**d**) A STEM image with the corresponding EDS elemental line-scan profiles of (**e**) Ag and (**f**) O for the graphene/welded Ag NWs region. (**g**) A STEM image of the graphene/welded Ag NWs region and (**h**) EELS C K-edge spectra of the graphene/welded Ag NWs region recorded with five spots (white arrows) near the edge of the Ag NWs corresponding to (**g**). (**i**) A suggest mechanism for the graphene/welded Ag NWs after 1 MeV HEBI.

From a different standpoint, we also focused on the structural evolution of graphene in graphene/welded Ag NWs hybrid films after HEBI. Resonant Raman spectra recorded at an excitation wavelength of 532 nm from graphene, graphene/Ag NWs, and graphene/welded Ag NWs are measured, indicating that graphene fingerprints including the D-, G-, and 2D-bands are observed, regardless of the film type (Supplementary Fig. S5). For graphene, the intensity ratios of the 2D- to G-bands and the D- to G-bands (I_D_/I_G_) correspond to 1.52 and 0.10, respectively, indicating the synthesis of monolayer graphene with a highly crystalline structure, which is confirmed by the SAED pattern (Fig. 1). Figure 3a,b represents the Raman G-band and I_D_/I_G_ maps for graphene extracted from the graphene/welded Ag NWs hybrid films. Interestingly, a significant increase in the intensity of the G-band for graphene is observed in the graphene/Ag NWs region (yellow and red circles) compared with that in the graphene-only region (green circle). This phenomenon can be explained by the effect of the Ag NWs-driven surface enhanced Raman scattering^30^. The I_D_/I_G_ map reveals that the relative intensity of the D-band for graphene significantly increases in the graphene/Ag NWs region. Figure 3c shows the D- and G-bands spectra from graphene in graphene-only region, graphene/welded Ag NWs region, and graphene/Ag NWs region, clearly indicating that the intensity of the D- and D′-bands increases in the graphene/welded Ag NWs region. This HEBI-induced defect generation phenomenon of the graphene in the welded Ag NWs can be explained as follows. Typically, lattice defects and thermal energy can be generated by knock-on collisions when carbon-based nanostructures are irradiated with energetic particles. Hence, the thermal energy generated by knock-on collision of irradiated electrons stimulates a localized chemical reaction between the atmospheric oxygen or reactive ozone and graphene, resulting in the formation of structural defects in graphene as well as the welding of Ag NWs. Namely, graphene plays the role of a sacrificial layer to suppress oxidation-induced structural damages in the welded Ag NWs. Energy-dispersive X-ray spectroscopy (EDS) and EELS were adopted to elucidate these phenomena. Figure 3d–f presents a scanning transmission electron microscopy (STEM) image with the corresponding EDS elemental line-scan profiles (black dotted arrow) of silver and oxygen for graphene/welded Ag NWs. These indicate that the intensity of the oxygen-related peak for graphene increases at the region of the welded Ag NWs. Figure 3g,h display a STEM image and the corresponding EELS C K-edge spectra of the graphene/welded Ag NWs recorded at five spots denoted by white arrows near the edge of the Ag NWs. These spectra indicate the presence of the C=C π* peak located at 290.2 eV and the C–C σ* peak at 299.2 eV for graphene. The C=C π* peak disappears when approaching the welded Ag NWs region, which supported by HEBI-stimulated local oxidation of graphene near the welded Ag NWs region, as depicted in Fig. 3i. The region of the sacrificial oxidation of graphene extends to a distance of only 60 nm from the welded Ag NWs junction, as confirmed by the EELS spectroscopy recorded at the five spots near the welded Ag NWs. This further supports the suggested methodology whereby the selective oxidation reaction of graphene is extremely localized to the desired area.

### Optical transmittance and bending tests of graphene/welded Ag NWs

**Figure 4:**
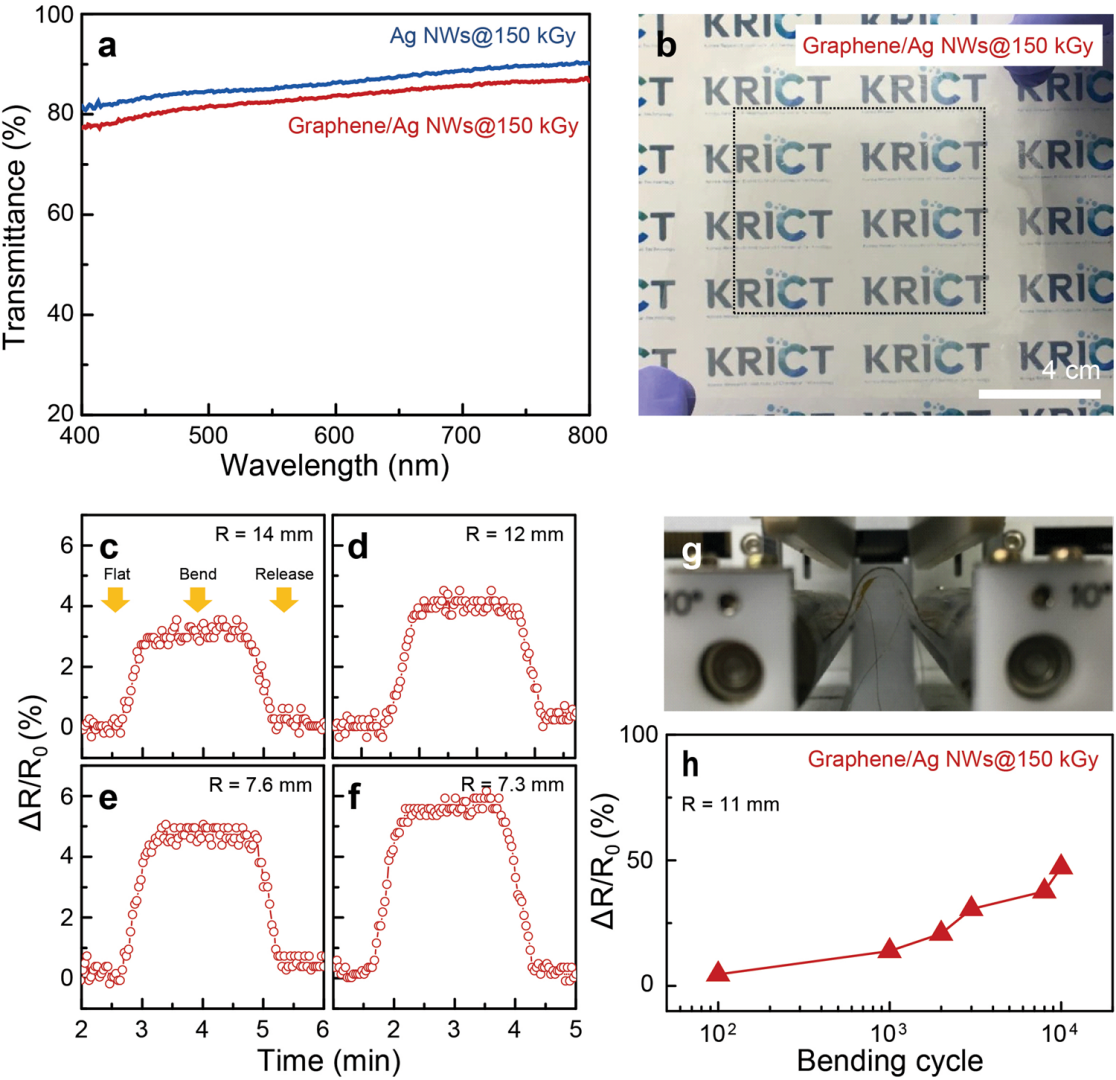
Optical transmittance and flexibility tests of the graphene/welded Ag NWs. (**a**) Optical transmittance at 550 nm wavelength for the Ag NWs and the graphene/Ag NWs on PET substrates after HEBI with a total flux of 150 kGy. (**b**) A photograph of the large-scale graphene/Ag NWs hybrid film onto a 10 cm × 10 cm PET substrate. Electrical resistance change of the Ag NWs/graphene on PET under bending processes with various bending radius of (**c**) R = 14 mm, (**d**) 12 mm, (**e**) 7.6 mm, and (**f**) 7.3 mm. (**g**) Bending process and (**h**) resistance variation of the Ag NWs/graphene formed by HEBI with a total flux of 150 kGy as a function of the bending cycles.

The optical transmittance at a wavelength of 550 nm of the Ag NWs and the graphene/Ag NWs after HEBI with a flux of 150 kGy corresponds to 83% (sheet resistance: 43.2 ± 5.9 Ohm/sq.) and 81% (12.9 ± 1.6 Ohm/sq.), respectively, as seen in Fig. 4a. Considering the opacity of the monolayer graphene (2.3 ± 0.1%), the optical transmittance of the hybrid films suggests the absence of residual carbon, which inevitably occurs during the PMMA-assisted wet transfer^8^. In addition, a photograph of the Ag NWs/graphene on PET (10 × 10 cm^2^ in size) is displayed in Fig. 4b. We mentioned earlier that our methodology for the formation of Ag NWs/graphene by HEBI with a flux of 150 kGy was conducted over a large area (90 × 90 cm^2^). However, the size of the hybrid film was restricted inevitably due to limitation of the CVD system for graphene growth. In order to assess the capability in flexible electronics, the variation in resistance (ΔR/R_0_) for the hybrid film is examined by adjusting the bending radius (r = 14, 12, 7.6, and 7.3 mm), as shown in Fig. 4c–f. With decreasing the bending radius, the relative changes in resistance of the hybrid films increase significantly. To examine the bending durability of the hybrid film formed by HEBI with a flux of 150 kGy, a variation in the resistance of the hybrid film with a bending radius of 11 mm as a function of bending cycles is explored, revealing that the variation in sheet resistance increases from ~4% to ~47% after 10^4^ cycles, as seen in Fig. 4g,h, which reflects excellent bending durability compared with that of previously reported graphene-Ag NWs hybrid films^31^.

## Conclusion

In summary, we have demonstrated the welding of Ag NWs encapsulated in graphene formed by flux-optimized HEBI under ambient conditions over a large area. After the HEBI-stimulated welding process with a flux of 150 kGy, the sheet resistance of the Ag NWs with graphene decreased significantly. Because of the encapsulation in graphene, the initial chemical states of the Ag NWs were invariant, and local oxidation of graphene occurred on behalf of the Ag NWs. We systematically studied that the effect of HEBI the sacrificial oxidation of graphene extended to only 60 nm away from the welded Ag NWs junction. Remarkably, the sheet resistance of the welded Ag NWs encapsulated in graphene after HEBI remained relatively unchanged after 85 days. It is envisaged that this methodology will result in an increased demand for transparent and flexible electrodes with high electrical stability.

## Methods

### Preparation of Ag NWs network and CVD-grown grapheme

First, a 0.5 wt.% solution of commercially available Ag NWs (Sigma Aldrich, diameter: 60 nm and length: 10 μm) in isopropyl alcohol was spin-coated onto a hydrophilic-treated SiO_2_ (300 nm)/Si(001) substrate at a rotational speed of 3000 rpm for 30 s, and then immediately heated to 150 °C for 5 min to remove the solvent. Next, graphene was synthesized using a conventional thermal chemical vapor deposition (TCVD) system. A 25 μm-thick Cu foil (Alfa Aesar, 99.8% purity) was utilized as a catalytic substrate for the synthesis of graphene. The Cu foil was loaded into the TCVD chamber and pre-annealed at 1050 °C with introducing H_2_ (200 sccm) under a pressure of ~3.6 Torr for 2 h in order to reduction and surface flattening of the Cu catalytic substrate. After the pre-annealing process, the graphene was immediately synthesized through the introduction of CH_4_ (2 sccm) and H_2_ (200 sccm) for 80 min. Following the growth of the graphene, the TCVD reactor was cooled down to room temperature with H_2_.

### HEBI of the graphene/Ag NWs hybrid films

The synthesized graphene was transferred onto the Ag NWs/SiO_2_ using a poly(methyl methacrylate) (PMMA)-assisted wet transfer technique. Nano-welding of the Ag NWs was accomplished through high energy electron beam irradiation (HEBI) of the graphene/Ag NWs hybrid films. A 1 MeV electron accelerator (EB-TECH Co. Ltd., Republic of Korea) was adopted for HEBI with the extraction window of 1200 mm in width. The total flux of the electron beam can be manipulated by adjusting the irradiation time at a dose rate of 33.3 kGy/sec. The samples were irradiated with a total flux of 30, 60, 90, 120, 150, 180, 200, 400, or 600 kGy under ambient conditions.

### Chemical and structural characterizations of the graphene/welded Ag NWs

The chemical identification of the hybrid films was conducted using X-ray photoelectron spectroscopy (XPS, Thermo scientific, ESCA Probe). The XPS spectra were recorded with a normal emission geometry using monochromatic Al Kα radiation (hν = 1486.6 eV) in an ultrahigh vacuum system (pressure: ~10^−9^ Torr) with a pass energy of 50.0 eV. The structural feature of the hybrid films was investigated using micro Raman spectroscopy (Renishaw, 514 nm, Ar^+^ ion laser). The structural characterization of the samples was carried out using scanning electron microscopy (SEM, S-4700, Hitach) and TEM (JEOL-2100F, JEOL) with electron energy loss spectroscopy (EELS, Gatan, Imaging filter model 607). The sheet resistance of the samples was measured by 4-point probe measurement system (CMT-SR1000N, Advanced Instrument Technology). The optical transmittance of the hybrid films was examined by UV-visible spectroscopy (UV-2501PC, SHIMADZU).

## Supplementary information

Supplementary Information
