# Supplementary material for: High energy electron beam stimulated nanowelding of silver nanowire networks encapsulated with graphene for flexible and transparent electrodes
Source: Sci Rep. 2019 Jun 28;9:9376. doi: 10.1038/s41598-019-45887-5 (PMC6598987; doi:10.1038/s41598-019-45887-5)
Supplement: Supplementary file 1 — Supplementary Information [file 41598_2019_45887_MOESM1_ESM.docx]

**High energy electron beam stimulated nanowelding of silver nanowire networks encapsulated with graphene for flexible and transparent electrodes**

Su Jin Lee^1^_,_ Young Bum Lee ^1^, Yi Rang Lim^1,2^, Jin Kyu Han^3^, In Su Jeon^1,4^, Garam Bae^1,5^, Yeoheung Yoon^1^, Wooseok Song^1,*^, Sung Myung^1^, Jongsun Lim^1^, Ki-Seok An^1^ & Sun Sook Lee^1,*^

^1^Thin Film Materials Research Center, Korea Research Institute of Chemical Technology, Yuseong Post Office Box 107, Daejeon 305-600, Republic of Korea.

^2^School of Electrical and Electronic Engineering, Yonsei University, 50 Yonsei-ro, Seodaemun-gu, Seoul 03722, Republic of Korea.

^3^Department of Energy Conversion and Storage, Technical University of Denmark, Frederiksborgvej 399, 4000, Roskilde, Denmark

^4^Department of Materials Science and Engineering, Hongik University, Seoul 121-791, Republic of Korea.

^5^Department of Physics, Sungkyunkwan University, Suwon, Gyeonggi-do 440-746, Republic of Korea.

Correspondence and requests for materials should be addressed to Wooseok Song and Sun Sook Lee (email: wssong@krict.re.kr, sunsukl@krict.re.kr).


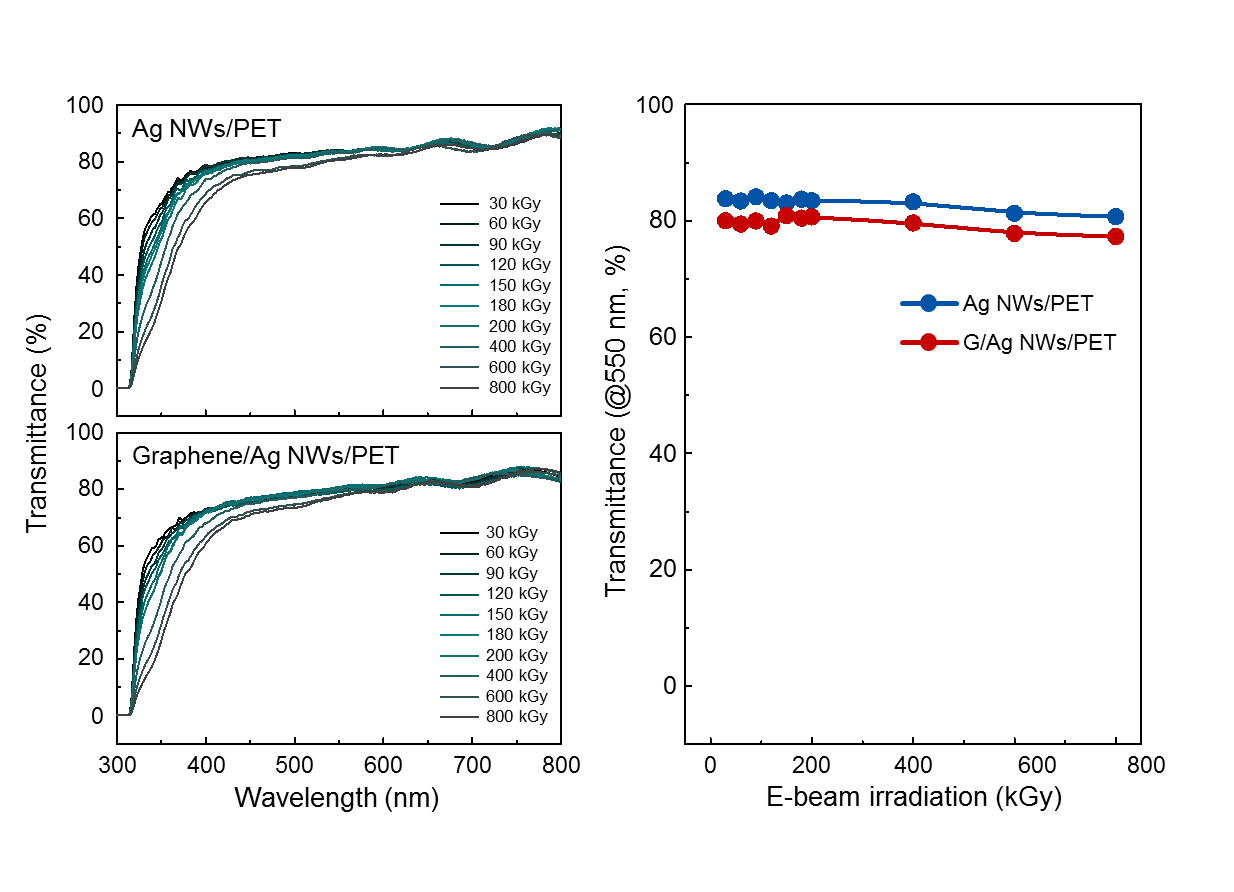


**Figure S1**. The optical transmittance analysis for the Ag NWs and the graphene/Ag NWs on PET substrates as a function of the HEBI flux.

| **Material** | **Transmittance**  **(%)** | | **Sheet resistance**  **(Ω/sq.)** | **Reference** |
| --- | --- | --- | --- | --- |
| Graphene/Ag NW | 92.8 | 34.4 | | [1] |
| Graphene/Ag NW | 85.0 | 74 | | [2] |
| Graphene/Ag NW | 93.0 | 200 | | [3] |
| Graphene/Ag NW | 88.9 | 19.9 | | [4] |
| Graphene/Ag NW | 80.0 | 86 | | [5] |
| rGO/Ag NW | 72.0 | 27 | | [6] |
| rGO/Ag-Cu NW | 77.6 | 10 | | [7] |
| rGO/Ag NW | 94.7 | 25 | | [8] |
| rGO/Ag NW | 92.0 | 17.3 | | [9] |
| rGO/Ag NW | 86.2 | 8 | | [10] |
| Graphene/Ag NW | **81** | **12.9** | | This work |

**Table S1**. Summary of the sheet resistance and optical transmittance for graphene/Ag NWs-based transparent electrodes


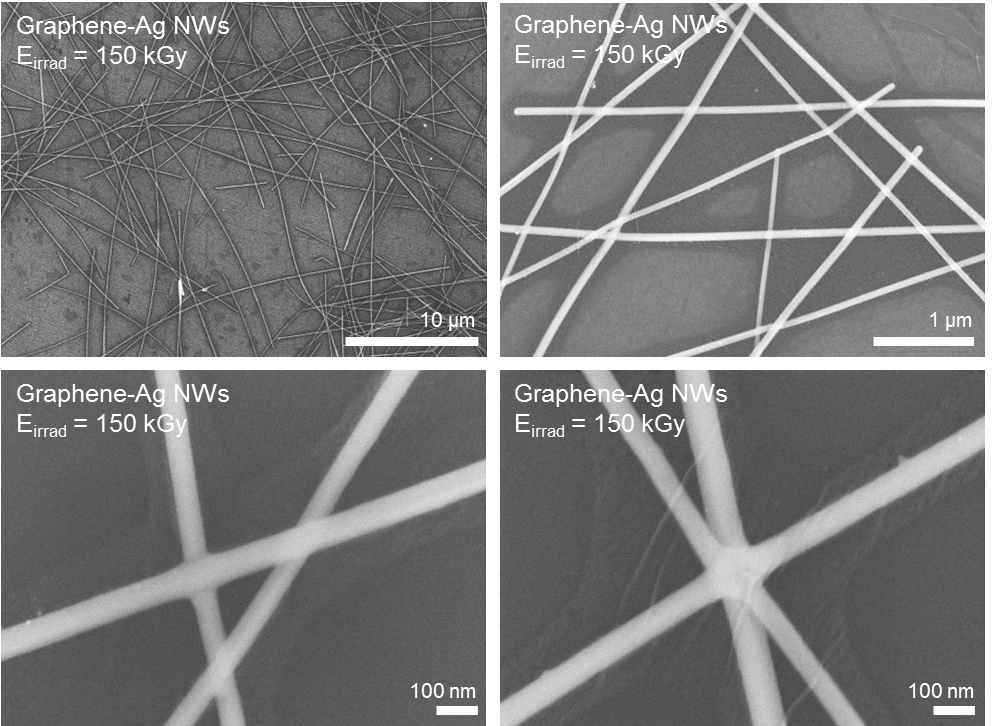


**Figure S2**. Representative low- and high-magnified SEM images of the graphene/welded Ag NWs after 1 MeV HEBI with a total flux of 150 kGy.

**
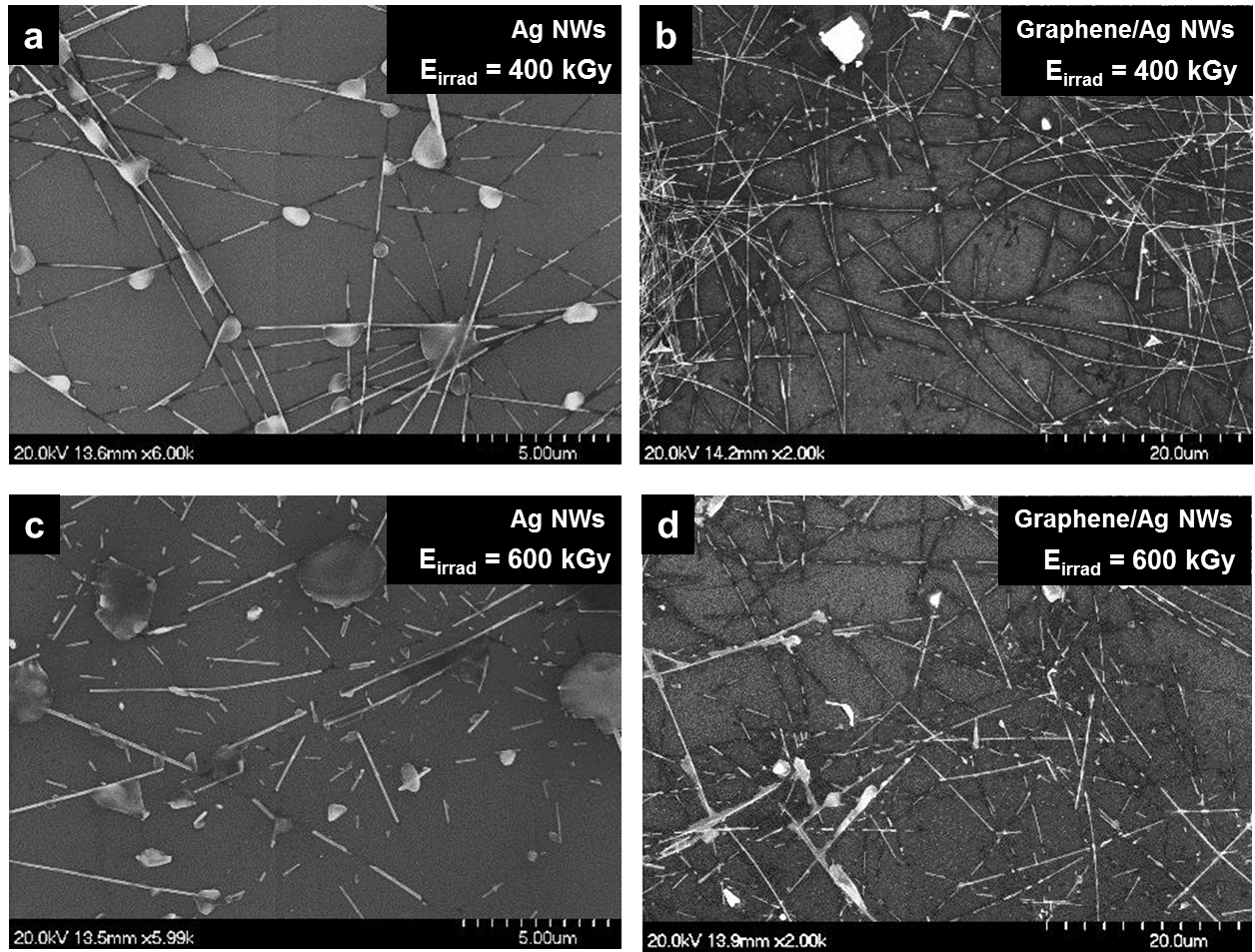
**

**Figure S3**. Representative SEM images of the welded Ag NWs and the graphene/welded Ag NWs after 1 MeV HEBI with a total flux (**a, b**) 400 kGy and (**c, d**) 600 kGy.


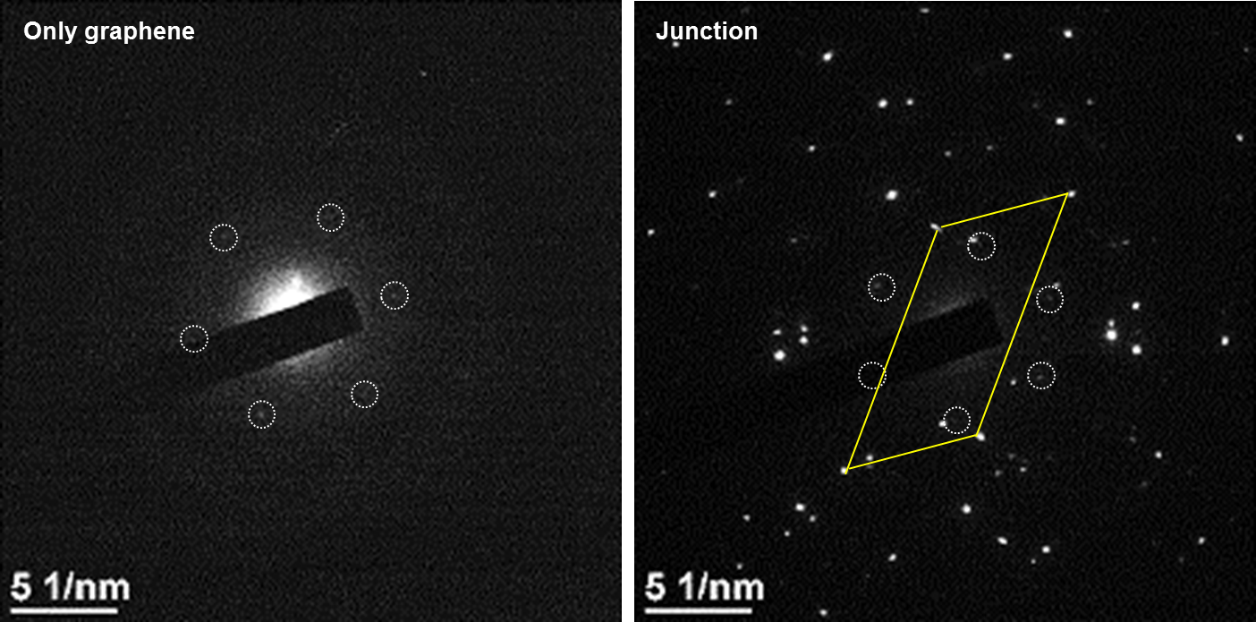


**Figure S4**. SAED patterns of the graphene/Ag NWs at (left) the graphene-only region and (right) the graphene/welded Ag NWs region corresponding to Fig. 1g (white and yellow dotted circles).

**
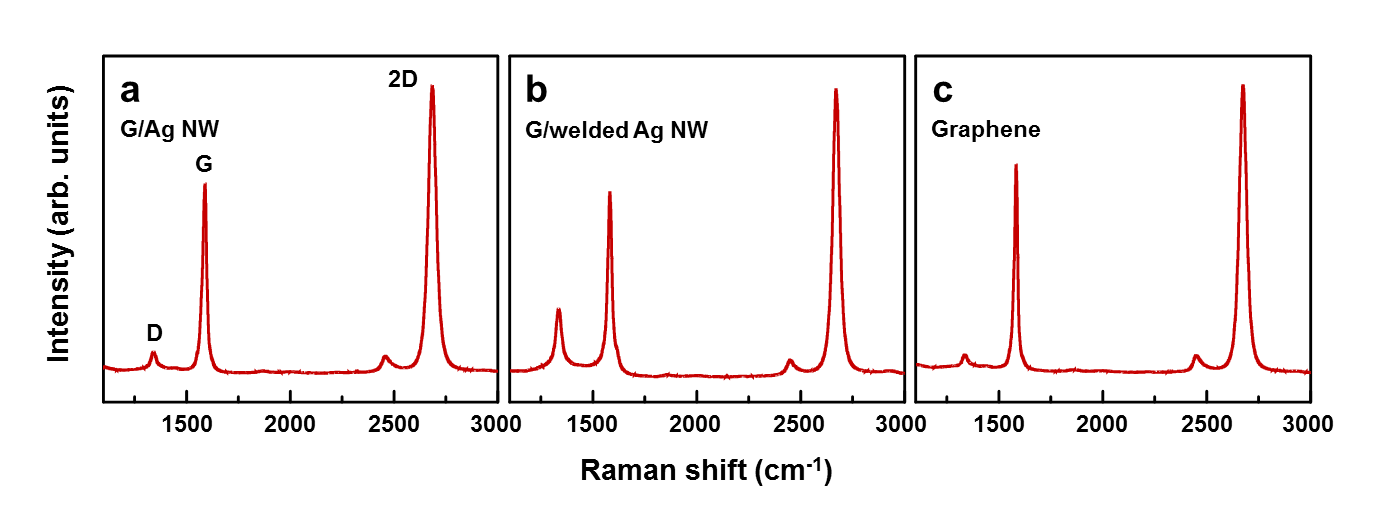
**

**Figure S5**. Resonant Raman spectra recorded at excitation wavelength of 532 nm for (**a**) the graphene/Ag NWs, (**b**) the graphene/welded Ag NWs, and (**c**) the graphene after HEBI with a total flux of 150 kGy.

**References**

1 Lee, D. *et al.* Highly stable and flexible silver nanowire–graphene hybrid transparent conducting electrodes for emerging optoelectronic devices. *Nanoscale* **5**, 7750-7755 (2013).

2 Hsiao, S.-T. *et al.* A highly electrically conductive graphene–silver nanowire hybrid nanomaterial for transparent conductive films. *Journal of Materials Chemistry C* **2**, 7284-7291 (2014).

3 Jurewicz, I. *et al.* Insulator‐conductor type transitions in graphene‐modified silver nanowire networks: a route to inexpensive transparent conductors. *Advanced Functional Materials* **24**, 7580-7587 (2014).

4 Lee, D., Lee, H., Ahn, Y. & Lee, Y. High-performance flexible transparent conductive film based on graphene/AgNW/graphene sandwich structure. *Carbon* **81**, 439-446 (2015).

5 Tien, H.-W. *et al.* Using self-assembly to prepare a graphene-silver nanowire hybrid film that is transparent and electrically conductive. *Carbon* **58**, 198-207 (2013).

6 Meenakshi, P., Karthick, R., Selvaraj, M. & Ramu, S. Investigations on reduced graphene oxide film embedded with silver nanowire as a transparent conducting electrode. *Solar Energy Materials Solar Cells* **128**, 264-269 (2014).

7 Kim, J. *et al.* Reduced graphene oxide wrapped core–shell metal nanowires as promising flexible transparent conductive electrodes with enhanced stability. *Nanoscale* **8**, 18938-18944 (2016).

8 Lai, Y.-T. & Tai, N.-H. One-step process for high-performance, adhesive, flexible transparent conductive films based on p-type reduced graphene oxides and silver nanowires. *ACS applied materials interfaces* **7**, 18553-18559 (2015).

9 Ahn, Y., Jeong, Y. & Lee, Y. Improved thermal oxidation stability of solution-processable silver nanowire transparent electrode by reduced graphene oxide. *ACS applied materials interfaces* **4**, 6410-6414 (2012).

10 Li, L., Li, W., Jiu, J. & Suganuma, K. Efficient assembly of high-performance reduced graphene oxide/silver nanowire transparent conductive film based on in situ light-induced reduction technology. *Applied Surface Science* **459**, 732-740 (2018).
